# Subclinical Hypothyroidism in Polycystic Ovary Syndrome: A Systematic Review and Meta-Analysis

**DOI:** 10.3389/fendo.2018.00700

**Published:** 2018-11-27

**Authors:** Xiaohong Ding, Lili Yang, Jian Wang, Rong Tang, Qianqian Chen, Jiexue Pan, Haiyan Yang, Xia Chen, Zimiao Chen, Liangshan Mu

**Affiliations:** ^1^Reproductive Medicine Center, The First Affiliated Hospital of Wenzhou Medical University, Wenzhou, China; ^2^Department of Radiology, The Second Affiliated Hospital and Yuying Children's Hospital of Wenzhou Medical University, Wenzhou, China; ^3^Department of Hand Surgery and Peripheral Neurosurgery, The First Affiliated Hospital of Wenzhou Medical University, Wenzhou, China; ^4^The Second School of Medicine, Wenzhou Medical University, Wenzhou, China; ^5^Department of Endocrinology, The First Affiliated Hospital of Wenzhou Medical University, Wenzhou, China

**Keywords:** subclinical hypothyroidism, thyroid, polycystic ovary syndrome, prevalence, meta-analysis

## Abstract

**Background:** The association between subclinical hypothyroidism (SCH) and polycystic ovary syndrome (PCOS) has been reported in several studies, but it is not well-recognized. The aim of this study was to evaluate the prevalence of SCH in women with PCOS.

**Methods:** An extensive literature search was conducted in PubMed, Embase, Web of Science, and Cochrane Library databases. All articles published before May 2018 was considered for eligibility. No language restrictions were implemented. The prevalence of SCH in PCOS was calculated by the meta-analysis to produce an odds ratio (OR) with 95% confidence interval (CI).

**Results:** A total of 6 studies including 692 PCOS patients and 540 controls were eligible for the meta-analysis. The combined odds ratio (OR) of SCH risk for women with PCOS compared with controls was 2.87 (95% CI = 1.82–9.92; *P* < 0.000001). The OR increased to 3.59 when limiting thyroid stimulating hormone (TSH) cut-off to ≥4 mIU/L.

**Conclusions:** Women with PCOS are more likely to develop SCH.

## Introduction

Polycystic ovary syndrome (PCOS) is one of the most common endocrine disorders characterized by anovulation, hyperandrogenism and polycystic ovaries, and affects up to 15–20% women of reproductive age (1). These patients are at risks of a range of metabolic and endocrinological disturbances including infertility, obesity, insulin resistance, and metabolic syndrome (2–4). In addition, there is also increasing evidence to suggest that PCOS links to the increased prevalence of thyroid diseases such as nodular goiter and autoimmune thyroiditis (5).

Primary hypothyroidism is a deficiency status in thyroid hormone production by the thyroid gland (6). It can cause a number of symptoms, such as poor ability to tolerate cold, tiredness, constipation, depression, and weight gain. Severity of hypothyroidism varies significantly, from transient and subclinical forms to severe cases. In fact, subclinical hypothyroidism (SCH), defined as an elevated TSH level in combination with normal T4 and free thyroxine (FT4) levels and lack of signs or symptoms of hypothyroidism, is more common than overt hypothyroidism (7). The prevalence of SCH is affected by geographic regions, ethnicity, and age in general population (8–10). Although SCH is a mild form, it also results in anovulatory cycles, sex hormone imbalances, subfertility, and adverse pregnancy outcomes (11–13), which are also features of women with PCOS. In addition, patients with SCH have increased metabolic risk of obesity, insulin resistance and hyperlipidemia similarly to those with PCOS (14, 15).

Considering that women with PCOS and SCH share the above mentioned features, we deduced that the presence of PCOS might be linked to the initiation and development of SCH. The prevalence of SCH in women with PCOS is variable, ranging from 11 to 36% (16, 17). To date, the overall prevalence of SCH in PCOS is limited by lack of large population-based data. In addition, no meta-analyses on this topic have been published. Hence, we aimed to conduct this systematic review and meta-analysis to evaluate the prevalence of SCH in women with PCOS.

## Methods

This systematic review and meta-analysis was designed according to the Preferred Reporting Items for Systematic Reviews and Meta-Analyses (PRISMA) statement (18) and Meta-analysis Of Observational Studies in Epidemiology (MOOSE) (19).

### Search strategy

An extensive literature search was conducted in PubMed, Embase, Web of Science and Cochrane Library databases. All articles published before May 2018 was considered for eligibility. No language restrictions were implemented. The search strategy is shown in Supplementary Table 1.

### Study selection criteria

Studies were selected according to the following criteria: (1) Studies defining PCOS clearly conforming to the Rotterdam Criteria or other compatible criteria were included; (2) Studies with clear data were included; (3) Studies on subjects having diseases other than PCOS and thyroid dysfunction, taking any other kind of medicine that could have influenced the test result were excluded; (4) Reviews, non-human studied and conference proceedings were excluded. Studies without control groups were only used to systematic review. Two reviewers scrutinized all articles identified by the searches independently. Discrepant opinions between the two reviewers were resolved by discussion and consultation with a third reviewer, if necessary.

### Data extraction

General study characteristics (name of the first author, year of publication, study location, type of study, number of women with and without PCOS), age of participants, diagnostic criteria for PCOS (Rotterdam or ESHRE/ASRM), definition of SCH, number of women with SCH in PCOS were extracted from each included study by using a standardized form. We used the available data for our analysis.

### Quality assessment

Quality of the included studies was assessed using the Cochrane Collaboration's tool. This tool evaluated seven domains, including random sequence generation, allocation concealment, blinding of participants and personnel, outcome assessment, incomplete outcome data, selective reporting, and other biases. Each item was classified as low risk, unclear risk, or high risk. Two reviewers evaluated the quality of included studies independently and discrepant opinions between the two reviewers were resolved by consensus. The possibility of publication bias was assessed by visual inspection of funnel plot.

### Statistical analysis

The software of Review Manager Version 5.3 was used to perform the effects by meta-analysis and to construct forest plot. The risk of SCH in women with PCOS compared with controls was expressed as odds ratio (OR) with its 95% confidence interval (CI). A forest plot summarized the results of all studies. The Cochran's chi-square-based Q statistic test and the *I*^2^ test were calculated to assess possible heterogeneity between the individual studies. An *I*^2^ values of 0–40, 40–60, and 60–100% were considered as unimportant heterogeneity, moderate heterogeneity and extensive heterogeneity, respectively.

## Results

### Study selection

**Figure 1:**
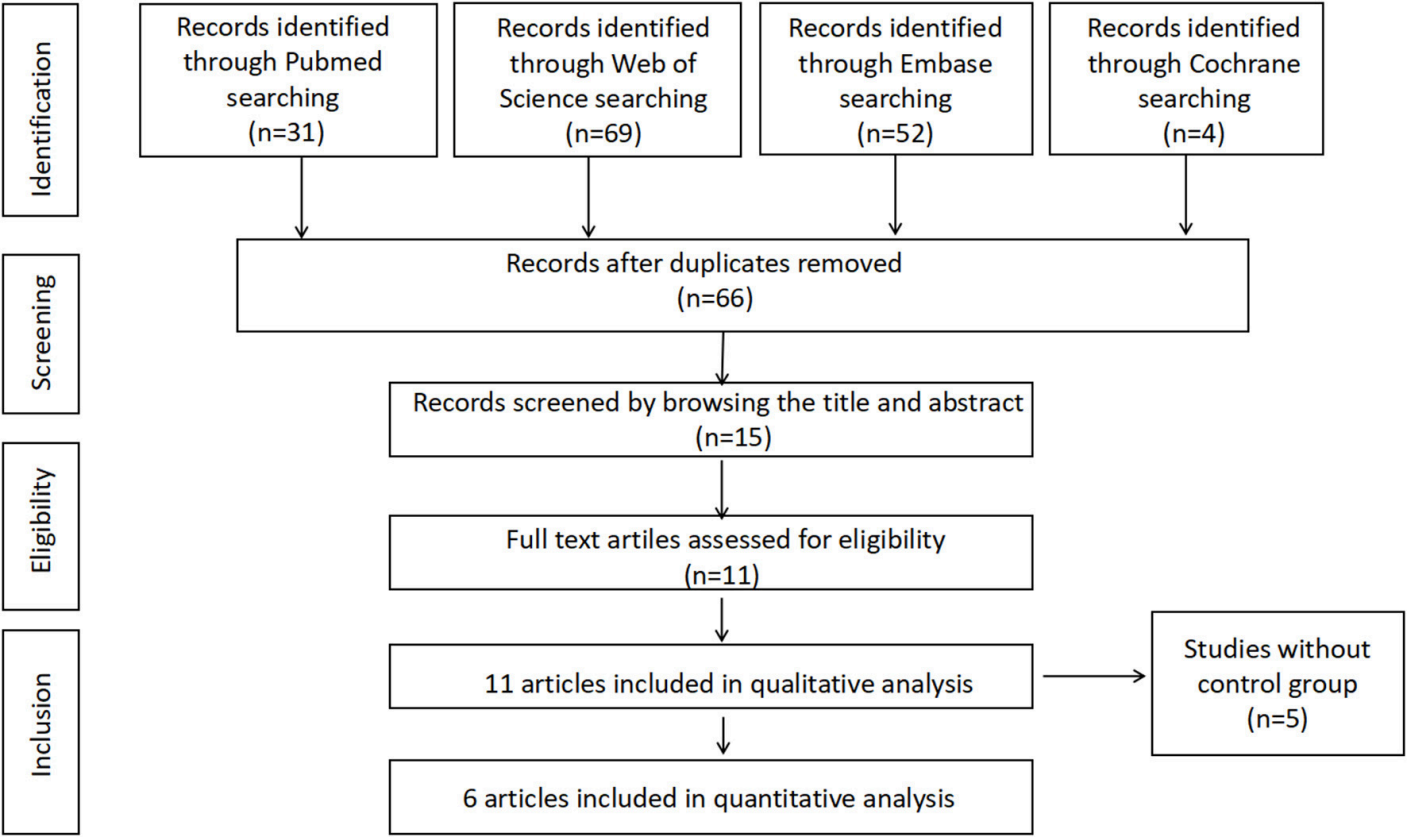
Flow chart of study inclusion in systematic review and meta-analysis.

Our search strategy identified 66 potential articles. Fifty-one studies were excluded after screening based on title or abstract, and 11 potentially relevant studies were assessed by reviewing the full-text article and finally included for the systematic review. Among these studies, 5 articles were excluded from the meta-analysis owing to lack of control groups. Finally, 6 studies including 692 subjects with PCOS and 540 controls met our inclusion criteria for the meta-analysis. Figure 1 presents the search strategy for study selection.

### Characteristics of included studies

General characteristics of the included studies are shown in Table 1. Of the 11 studies, 8 are cross-sectional design and 3 are case-control design. The majority of studies used the Rotterdam criteria for PCOS diagnosis and only one study used the ESHRE/ASRM criteria. Subjects included in the meta-analysis were from five different countries including Spain (17), India (22), Italy (20, 23), Brazil (21), and China (24), while those only included in systematic review were from America (25), India (26), China (27), Brazil (16), and Iran (28). Definition for SCH varied among studies, with TSH cut-off values ranging from 2.5 to 5 mIU/L. Among these studies, the prevalence of SCH in PCOS varied significantly, ranging from 11.3 to 36.6% (Table 2).

**Table 1:** General characteristics of studies included in the systematic review.

**Study**	**Type**	**Region**	**PCOS diagnosis criteria**	**Definition for SCH**	**Age (PCOS vs. controls)**
(17)	Case-control study	Spain	Rotterdam 2003 criteria	TSH ≥ 4.2 mIU/L	24.5 ± 6.7; 26.3 ± 7.4
(20)	Case-control study	Italy	ESHRE/ASRM consensus	TSH > 2.5 mIU/L	32.2 ± 6.5; 36.7 ± 6.5
(21)	Cross-sectional study	Brazil	Rotterdam criteria	TSH: 4.5–10 mIU/L, normal FT4 levels (0.9–1.8 ng/dl)	27.8 ± 6.9; 33.5 ± 5.7
(22)	Cross-sectional study	India	Rotterdam criteria	TSH > 4.25 mIU/L	22.7 ± 5.30; 26.3 ± 7.4
(23)	Cross-sectional study	Italy	Rotterdam criteria	TSH > 2.5 mIU/L	18–36; 18–36
(24)	Case-control study	China	Rotterdam criteria	TSH > 4.25 mIU/L, normal T3 and T4 levels	27.4 ± 5.4; 23.3 ± 4.1
(25)	Cross-sectional study	America	Rotterdam criteria	TSH > 2.5 mIU/L	29.5; /
(26)	Cross-sectional study	India	Rotterdam criteria	/	19 ± 4.84; /
(16)	Cross-sectional study	China	Rotterdam criteria	TSH > 5 mIU/L	26.72 ± 5.43; /
(27)	Cross-sectional cohort study	Brazil	Rotterdam criteria	TSH: 4.5–10 mIU/L	24 ± 5.8; /
(28)	Cross sectional study	Iran	Rotterdam criteria	TSH > 3.75 mIU/L, normal levels of FT3 and FT4	26 ± 4.2; /

**Table 2:** The distribution of subclinical hypothyroidism in PCOS and controls.

**Study**	**SCH/PCOS(%)**	**SCH/controls(%)**
(17)	52/142 (36.6)	7/52 (13.5)
(20)	51/151 (33.8)	36/155 (23.2)
(21)	11/65 (16.9)	4/65 (6.2)
(22)	18/80 (22.5)	7/80 (8.8)
(23)	22/154 (14.3)	1/88 (1.1)
(24)	27/100 (27.0)	8/100 (8.0)
(25)	30/137 (21.9)	/
(26)	16/60 (26.6)	/
(16)	60/428 (14.0)	/
(27)	19/168 (11.3)	/
(28)	19/75 (25.3)	/

*No., number; SCH, subclinical hypothyroidism; TSH: thyroid stimulating hormone; FT3: Free triiodothyronine; FT4: free thyroxine*.

### Quality assessment

**Figure 2:**
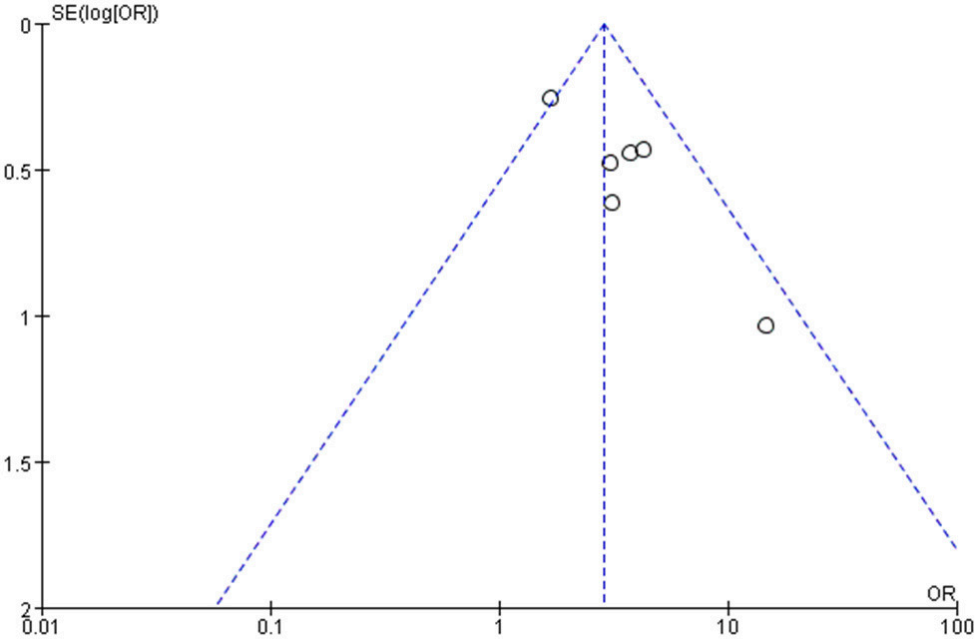
Funnel plot of the studies.

The risk of bias for the six studies in meta-analysis was evaluated using the Cochrane Collaboration's tool. All studies showed a low risk of bias in incomplete outcome data and selective reporting but the evaluation of the random sequence generation and allocation concealment were not clear. Because of the small sample population, two studies have other bias (Supplementary Figure 1). The funnel plot showed reasonable symmetry, with no evidence of publication bias (Figure 2). It is thus concluded that the meta-analysis finding is robust.

### Subclinical hypothyroidism in PCOS

**Figure 3:**
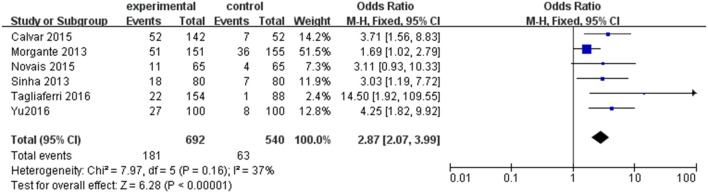
Forest plot of the prevalence of SCH in women with and without PCOS. Details are given for events, number of included subjects, and odds ratio for each study as well as the overall events, subjects numbers, and odds ratio given in bold in the “Total” row. CI, confidence interval; df, degrees of freedom; M-H, Mantel-Haenszel; SCH, subclinical hypothyroidism.

Six individual studies were included to compare the prevalence of SCH between PCOS and controls. The meta-analysis showed that the combined OR of SCH risk for women with PCOS compared with controls was 2.87 (95% CI = 1.82–9.92; *P* < 0.000001; Figure 3). There was low heterogeneity among these studies (*P* = 0.16; *I*^2^ = 37).

**Figure 4:**
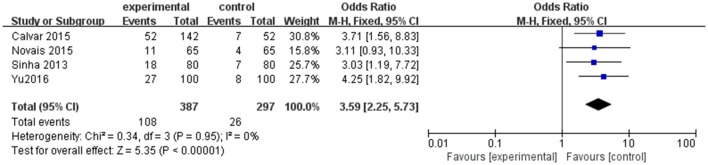
Subgroup analysis for SCH and PCOS when TSH cut off is higher than 4.0 mIU/mL. Details are given for events, number of included subjects, and odds ratio for each study as well as the overall events, subjects numbers, and odds ratio given in bold in the “Total” row. CI, confidence interval; df, degrees of freedom; M-H, Mantel-Haenszel; SCH, subclinical hypothyroidism.

However, as shown in Table 1, the cut-off for TSH to establish the diagnosis of SCH is different, which is one of the possible limitations to our results. Thus, a subgroup analysis including four studies in which TSH upper limit was more than 4.0 mIU/l was performed. An evident difference between women with PCOS and controls was found in the composite endpoint (OR 3.59; 95% CI = 2.25–5.73; *P* < 0.000001; Figure 4). There was little heterogeneity among four studies (*P* = 0.95; *I*^2^ = 0).

## Discussion

To our knowledge, this was the first systematic review and meta-analysis aimed at quantifying the relationship between SCH and PCOS. This meta-analysis including six studies showed that women with PCOS had 2.87 times the odds of having SCH than controls, suggesting PCOS might be a risk factor for SCH.

Over the past decades, a large number of studies had investigated the prevalence of SCH in PCOS. Since the prevalence of SCH differs from geographic region, ethnicity or age, the results of studies were inconsistent. Only 14.3% of participants had SCH in the Italian PCOS population (23), whereas 27.0% of participants had SCH in China (24). The reported prevalence of SCH in the Indian PCOS population was nearly 22.5% (22), which was higher than that in Brazil (21). Although a recent study showed that SCH does not increase the risk of PCOS in obese women of reproductive age (29), it is recognized by most researchers that PCOS exacerbates the development of SCH.

Several possible mechanisms for the increased prevalence of SCH in PCOS have been inferred. First, the effect of PCOS on the SCH is likely to be mediated by obesity and insulin resistance. Excessive body weight seems to promote this interplay (23). In addition, there was no difference in the mean values of all endocrine and metabolic parameters tested in the presence or absence of SCH with PCOS. However, abnormal FPG levels and insulin resistance were more likely in women who had SCH than in women without SCH independently of age and BMI (25). What is critical is that SCH is associated with insulin resistance (30). Second, compromised immune system is likely to be a cause of the interaction between SCH and PCOS since SCH can result from autoimmune thyroiditis (31). Normally, estrogen's immune stimulatory activity is neutralized by anti-inflammatory actions of progesterone levels. However, progesterone level is near zero in PCOS because of anovulatory cycles (32). As a result, estrogen overstimulates the immune system, leading to high incidence of autoimmune diseases (33). Third, the strong direct interaction between thyroid and ovary has been implied by experiments both in humans and animals. For example, thyroglobulin and TSH receptor are detected in bovine luteal cells by immunohistochemistry suggesting that the luteal cells of mature corpora lutea may be involved in the synthesis of thyroid hormones (34).

In the long term, PCOS women with SCH might have increased risk of developing a variety of diseases. Total cholesterol (TC), triglyceride (TG) and fasting glucose were higher in PCOS with SCH (35, 36), thus they are more likely to exhibit hyperlipidemia, impaired glucose metabolism, and cardiovascular diseases (24, 37). SCH may also contribute to the psychological co-morbidities in women with PCOS, such as anxiety and depression, since the strong association between SCH, PCOS and depressive symptoms has been reported, respectively (38, 39). Besides, SCH during pregnancy could lead to multiple adverse maternal and neonatal outcomes, including premature rupture of membranes and neonatal death (40).

Therefore, greater awareness is needed for PCOS women with SCH. Metformin may be a beneficial choice for PCOS women with SCH. A significantly reduction in serum TSH levels was observed in patients with SCH after treatment with metformin and the effect was not related to its dose. Several mechanisms have hypothesized for explaining this effect: (1) a change in the affinity or number of TSH receptors; (2) an increase in the central dopaminergic tone; or (3) an interaction between metformin and TSH (41). Meanwhile, metformin also plays a role in improving the ovulation rate and reproductive outcomes in women with PCOS (42). In addition, it was reported that levothyroxine replacement therapy can improve clinical pregnancy outcome in women with SCH undergoing assisted reproductive technology, not only significantly increasing delivery rate but also lowering miscarriage rate (43). Besides, levothyroxine therapy is associated with a decreased risk of low birth weight (44).

## Limitations

Our literature search was comprehensive, and we did not apply any restrictions on language to limit our ability to assess the relationship between SCH and PCOS. It is undeniable that several limitations present in our meta-analysis. First, the included studies were not restricted to specific range of age and were designed as naturalistic analysis (cross-sectional and case-control studies) with different data collection. Our sample capacity was still too small to avoid random error and most participants included were from clinics or hospitals. Thus, the PCOS groups in this review may be over-represented by those with more severe symptom. Furthermore, there may be possible bias due to the heterogeneity in terms of SCH definition (based on TSH upper limit) and PCOS diagnosis. In spite of these limitations, the present meta-analysis has increased the statistical power by pooling the results of single studies. Therefore, the total number of the subjects was sufficiently large to support our conclusion.

## Conclusion

In summary, this systematic review and meta-analysis demonstrated that PCOS was strongly associated with an increased risk of SCH. Further studies are needed to explore the potential pathways and focus on whether SCH could be improved by treating PCOS.

## Author contributions

XD and LY were engaged in analysis and interpretation of data, prepared and drafted manuscript. JW, RT, and QC were involved in article revision. JP was involved in the acquisition of data. HY and ZC were involved in execution. XC contributed to conception and design of study. LM contributed to conception, study design, and article revision.

### Conflict of interest statement

The authors declare that the research was conducted in the absence of any commercial or financial relationships that could be construed as a potential conflict of interest.

## Supplementary material

Supplementary Table 1 Search strategy and selected keywords.

Supplementary Figure 1 Risk of bias summary: review authors' judgments about each risk of bias item for each included study.

## References

[1] March WA Moore VM Willson KJ Phillips DI Norman RJ Davies MJ. The prevalence of polycystic ovary syndrome in a community sample assessed under contrasting diagnostic criteria. Hum Reprod. (2010) 25:544–51. 10.1093/humrep/dep399 19910321

[2] Morales AJ Laughlin GA Butzow T Maheshwari H Baumann G Yen SS. Insulin, somatotropic, and luteinizing hormone axes in lean and obese women with polycystic ovary syndrome: common and distinct features. J Clin Endocrinol Metab. (1996) 81:2854–64. 8768842 10.1210/jcem.81.8.8768842

[3] Carmina E Lobo RA. Polycystic ovary syndrome (PCOS): arguably the most common endocrinopathy is associated with significant morbidity in women. J Clin Endocrinol Metab. (1999) 84:1897–9. 10.1210/jcem.84.6.5803 10372683

[4] Moran LJ Norman RJ Teede HJ. Metabolic risk in PCOS: phenotype and adiposity impact. Trends Endocrinol Metab. (2015) 26:136–43. 10.1016/j.tem.2014.12.003 25591984

[5] Duran C Basaran M Kutlu O Kucukaydin Z Bakdik S Burnik FS. Frequency of nodular goiter and autoimmune thyroid disease in patients with polycystic ovary syndrome. Endocrine (2015) 49:464–9. 10.1007/s12020-014-0504-7 25522724

[6] Diaz A Lipman Diaz EG. Hypothyroidism. Pediatr Rev. (2014) 35:336–47; quiz 348–39. 10.1542/pir.35-8-336 25086165

[7] Teng W Shan Z Patil-Sisodia K Cooper DS. Hypothyroidism in pregnancy. Lancet Diabetes Endocrinol. (2013) 1:228–37. 10.1016/S2213-8587(13)70109-8 24622371

[8] Hollowell JG Staehling NW Flanders WD Hannon WH Gunter EW Spencer CA. Serum TSH, T(4), and thyroid antibodies in the United States population (1988 to 1994): National Health and Nutrition Examination Survey (NHANES III). J Clin Endocrinol Metab. (2002) 87:489–99. 10.1210/jcem.87.2.8182 11836274

[9] Surks MI Hollowell JG. Age-specific distribution of serum thyrotropin and antithyroid antibodies in the US population: implications for the prevalence of subclinical hypothyroidism. J Clin Endocrinol Metab. (2007) 92:4575–82. 10.1210/jc.2007-1499 17911171

[10] Dittrich R Kajaia N Cupisti S Hoffmann I Beckmann MW Mueller A. Association of thyroid-stimulating hormone with insulin resistance and androgen parameters in women with PCOS. Reprod Biomed Online (2009) 19:319–25. 10.1016/S1472-6483(10)60165-4 19778476

[11] Cho MK. Thyroid dysfunction and subfertility. Clin Exp Reprod Med. (2015) 42:131–5. 10.5653/cerm.2015.42.4.131 26816871 PMC4724596

[12] Barisic T Mandic V Vasilj A Tiric D. Higher levels of thyrotropin in pregnancy and adverse pregnancy outcomes. J Matern Fetal Neonatal Med. (2018). 1–6. 10.1080/14767058.2018.1451509 29540085

[13] Yang J Liu Y Liu H Zheng H Li X Zhu L. Associations of maternal iodine status and thyroid function with adverse pregnancy outcomes in Henan Province of China. J Trace Elem Med Biol. (2018) 47:104–10. 10.1016/j.jtemb.2018.01.013 29544795

[14] Ganie MA Laway BA Wani TA Zargar MA Nisar S Ahamed F. Association of subclinical hypothyroidism and phenotype, insulin resistance, and lipid parameters in young women with polycystic ovary syndrome. Fertil Steril. (2011) 95:2039–43. 10.1016/j.fertnstert.2011.01.149 21333983

[15] Celik C Abali R Tasdemir N Guzel S Yuksel A Aksu E. Is subclinical hypothyroidism contributing dyslipidemia and insulin resistance in women with polycystic ovary syndrome? Gynecol Endocrinol. (2012) 28:615–8. 10.3109/09513590.2011.650765 22329744

[16] Benetti-Pinto CL Berini Piccolo VRS Garmes HM Teatin Juliato CR. Subclinical hypothyroidism in young women with polycystic ovary syndrome: an analysis of clinical, hormonal, and metabolic parameters. Fertil Steril. (2013) 99:588–92. 10.1016/j.fertnstert.2012.10.006 23103018

[17] Calvar CE Bengolea SV Deutsch SI Hermes R Ramos G Loyato M. [High frequency of thyroid abnormalities in polycystic ovary syndrome]. Medicina (B Aires) (2015) 75:213–7. 26339875

[18] Moher D Liberati A Tetzlaff J Altman DG Preferred reporting items for systematic reviews and meta-analyses: the PRISMA statement. PLoS Med. (2009) 6:e1000097 10.1371/journal.pmed.1000097 19621072 PMC2707599

[19] Stroup DF Berlin JA Morton SC Olkin I Williamson GD Rennie D. Meta-analysis of observational studies in epidemiology: a proposal for reporting. JAMA (2000) 283:2008–12. 10.1001/jama.283.15.2008 10789670

[20] Morgante G Musacchio MC Orvieto R Massaro MG De Leo V. Alterations in thyroid function among the different polycystic ovary syndrome phenotypes. Gynecol Endocrinol. (2013) 29:967–9. 10.3109/09513590.2013.829445 23957782

[21] Novais Jde S Benetti-Pinto CL Garmes HM Jales RM Juliato CR Polycystic ovary syndrome and chronic autoimmune thyroiditis. Gynecol Endocrinol. (2015) 31:48–51. 10.3109/09513590.2014.958990 25211537

[22] Sinha U Sinharay K Saha S Longkumer TA Baul SN Pal SK. Thyroid disorders in polycystic ovarian syndrome subjects: a tertiary hospital based cross-sectional study from Eastern India. Indian J Endocrinol Metab. (2013) 17:304–9. 10.4103/2230-8210.109714 23776908 PMC3683210

[23] Tagliaferri V Romualdi D Guido M Mancini A De Cicco S Di Florio C. The link between metabolic features and TSH levels in polycystic ovary syndrome is modulated by the body weight: an euglycaemic-hyperinsulinaemic clamp study. Eur J Endocrinol. (2016) 175:433–41. 10.1530/EJE-16-0358 27511825

[24] Yu Q Wang J-B. Subclinical hypothyroidism in PCOS: impact on presentation, insulin resistance, and cardiovascular risk. BioMed Res Int. (2016) 2016:1–7. 10.1155/2016/2067087 27478827 PMC4960326

[25] Bedaiwy MA Abdel-Rahman MY Tan J AbdelHafez FF Abdelkareem AO Henry D. Clinical, hormonal, and metabolic parameters in women with subclinical hypothyroidism and polycystic ovary syndrome: a cross-sectional study. J Womens Health (Larchmt) (2018) 27:659–64. 10.1089/jwh.2017.6584 29620956

[26] Ganvir S Sahasrabuddhe A Pitale S Thyroid function tests in polycystic ovarian syndrome. Natl J Physiol Pharm Pharmacol. (2017) 7:1 10.5455/njppp.2017.7.0926503102016

[27] Huang R Zheng J Li S Tao T Liu W. Subclinical hypothyroidism in patients with polycystic ovary syndrome: distribution and its association with lipid profiles. Eur J Obstet Gynecol Reprod Biol. (2014) 177:52–6. 10.1016/j.ejogrb.2014.04.013 24768234

[28] Enzevaei A Salehpour S Tohidi M Saharkhiz N. Subclinical hypothyroidism and insulin resistance in polycystic ovary syndrome: is there a relationship? Iran J Reprod Med. (2014) 12:481–6. 25114670 PMC4126252

[29] Zhang B Wang J Shen S Liu J Sun J Gu T. Subclinical hypothyroidism is not a risk factor for polycystic ovary syndrome in obese women of reproductive age. Gynecol Endocrinol. (2018). 34:875–9. 10.1080/09513590.2018.1462319 29658805

[30] Vyakaranam S Vanaparthy S Nori S Palarapu S Bhongir AV. Study of insulin resistance in subclinical hypothyroidism. Int J Health Sci Res. (2014) 4:147–53. 25580384 PMC4286301

[31] Effraimidis G Wiersinga WM. Mechanisms in endocrinology: autoimmune thyroid disease: old and new players. Eur J Endocrinol. (2014) 170:R241–52. 10.1530/EJE-14-0047 24609834

[32] Petrikova J Lazurova I Yehuda S. Polycystic ovary syndrome and autoimmunity. Eur J Intern Med. (2010) 21:369–71. 10.1016/j.ejim.2010.06.008 20816585

[33] Angstwurm MW Gartner R Ziegler-Heitbrock HW. Cyclic plasma IL-6 levels during normal menstrual cycle. Cytokine (1997) 9:370–4. 10.1006/cyto.1996.0178 9195137

[34] Mutinati M Desantis S Rizzo A Zizza S Ventriglia G Pantaleo M. Localization of thyrotropin receptor and thyroglobulin in the bovine corpus luteum. Anim Reprod Sci. (2010) 118:1–6. 10.1016/j.anireprosci.2009.05.019 19553036

[35] Pergialiotis V Konstantopoulos P Prodromidou A Florou V Papantoniou N Perrea DN. MANAGEMENT OF ENDOCRINE DISEASE: the impact of subclinical hypothyroidism on anthropometric characteristics, lipid, glucose and hormonal profile of PCOS patients: a systematic review and meta-analysis. Eur J Endocrinol. (2017) 176:R159–66. 10.1530/EJE-16-0611 28007842

[36] de Medeiros SF de Medeiros MAS Ormond CM Barbosa JS Yamamoto MMW Subclinical hypothyroidism impact on the characteristics of patients with polycystic ovary syndrome. A meta-analysis of observational studies. Gynecol Obstet Invest. (2018) 83:105–15. 10.1159/000485619 30025406

[37] Pei YJ Wang AM Zhao Y Yan L Li M White RE. Studies of cardiovascular risk factors in polycystic ovary syndrome patients combined with subclinical hypothyroidism. Gynecol Endocrinol. (2014) 30:553–6. 10.3109/09513590.2013.829443 24884959

[38] Teede H Deeks A Moran L. Polycystic ovary syndrome: a complex condition with psychological, reproductive and metabolic manifestations that impacts on health across the lifespan. BMC Med. (2010) 8:41. 10.1186/1741-7015-8-41 20591140 PMC2909929

[39] Demartini B Ranieri R Masu A Selle V Scarone S Gambini O. Depressive symptoms and major depressive disorder in patients affected by subclinical hypothyroidism: a cross-sectional study. J Nerv Ment Dis. (2014) 202:603–7. 10.1097/NMD.0000000000000168 25010109

[40] Maraka S Ospina NM O'Keeffe DT Espinosa De Ycaza AE Gionfriddo MR Erwin PJ. Subclinical hypothyroidism in pregnancy: a systematic review and meta-analysis. Thyroid (2016) 26:580–90. 10.1089/thy.2015.0418 26837268 PMC4827301

[41] Rotondi M Cappelli C Magri F Botta R Dionisio R Iacobello C. Thyroidal effect of metformin treatment in patients with polycystic ovary syndrome. Clin Endocrinol. (2011) 75:378–81. 10.1111/j.1365-2265.2011.04042.x 21521311

[42] Tang T Lord JM Norman RJ Yasmin E Balen AH Insulin-sensitising drugs (metformin, rosiglitazone, pioglitazone, D-chiro-inositol) for women with polycystic ovary syndrome, oligo amenorrhoea and subfertility. Cochrane Database Syst Rev. (2012) Cd003053 10.1002/14651858.CD003053.pub5 22592687

[43] Velkeniers B Van Meerhaeghe A Poppe K Unuane D Tournaye H Haentjens P. Levothyroxine treatment and pregnancy outcome in women with subclinical hypothyroidism undergoing assisted reproduction technologies: systematic review and meta-analysis of RCTs. Hum Reprod Update (2013) 19:251–8. 10.1093/humupd/dms052 23327883

[44] Maraka S Singh Ospina NM O'Keeffe DT Rodriguez-Gutierrez R Espinosa De Ycaza AE Wi CI. Effects of levothyroxine therapy on pregnancy outcomes in women with subclinical hypothyroidism. Thyroid (2016) 26:980–6. 10.1089/thy.2016.0014 27112035 PMC4939379

