# Supplementary material for: Subclinical Hypothyroidism in Polycystic Ovary Syndrome: A Systematic Review and Meta-Analysis
Source: Front Endocrinol (Lausanne). 2018 Nov 27;9:700. doi: 10.3389/fendo.2018.00700 (PMC6277795; doi:10.3389/fendo.2018.00700)
Supplement: Supplementary Table 1 — Search strategy and selected keywords. [file Table_1.doc]

Supplementary Material

# Title: Subclinical hypothyroidism in polycystic ovary syndrome: a systematic review and meta-analysis

Xiaohong Ding^1†^, Lili Yang^2†^, Jian Wang^3^, Rong Tang^4^, Qianqian Chen^1^, Jiexue Pan^1^, Haiyan Yang^1^, Xia Chen^1^, Zimiao Chen^5^*and Liangshan Mu^1^*

^1^ Reproductive Medicine Center, The First Affiliated Hospital of Wenzhou Medical University, Wenzhou 325000, People’s Republic of China;

^2^ Department of Radiology, The Second Affiliated Hospital and Yuying Children’s Hospital of Wenzhou Medical University, Wenzhou 325000, People’s Republic of China;

^3^ Department of Hand Surgery and Peripheral Neurosurgery, The First Affiliated Hospital of Wenzhou Medical University, Wenzhou 325000, People’s Republic of China ;

^4^ The Second School of Medicine, Wenzhou Medical University, Wenzhou 325000, People’s Republic of China ;

^5^ Department of Endocrinology, The First Affiliated Hospital of Wenzhou Medical University, Wenzhou 325000, People’s Republic of China;

^†^, These two authors contributed equally to this article.

*, These two authors are corresponding authors.

**Correspondence to:** Liangshan Mu, M.D., Reproductive Medicine Center, The First Affiliated Hospital of Wenzhou Medical University, No.96 Fuxue Road, Wenzhou 325000, People’s Republic of China; E-mail: [liangshanmu@hotmail.com](mailto:liangshanmu@hotmail.com); Zimiao Chen, M.D., Department of Endocrinology, The First Affiliated Hospital of Wenzhou Medical University, No.96 Fuxue Road, Wenzhou 325000, People’s Republic of China; E-mail: zimiaochen@163.com;

**Supplementary Table I : Search strategy and selected keywords**

((Polycystic Ovary Syndrome OR Stein Leventhal Syndrome OR Sclerocystic Ovari* OR PCOS OR polycystic ovary disease OR PCOD)) AND (subclinical hypothyroidism OR SHT OR SCH). The references of the identified studies were also checked.
