# Supplementary figures and images for: Subclinical Hypothyroidism in Polycystic Ovary Syndrome: A Systematic Review and Meta-Analysis
Source: Front Endocrinol (Lausanne). 2018 Nov 27;9:700. doi: 10.3389/fendo.2018.00700 (PMC6277795; doi:10.3389/fendo.2018.00700)

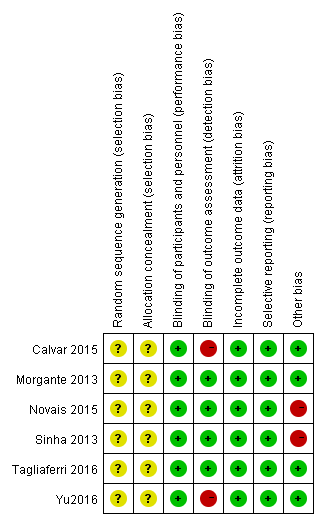

Supplement: Supplementary Figure 1 — Risk of bias summary: review authors' judgments about each risk of bias item for each included study. [file Image_1.tif]
